# The protective influence of orange essential oil nano-emulsion on growth performance, antioxidant activity, immune status, inflammatory response, and hepato-renal function in heat-stressed growing rabbits

**DOI:** 10.1007/s11250-026-05005-w

**Published:** 2026-04-17

**Authors:** Hamada A. Areda, Mahmoud A. E. Hassan, Eman M. Embaby, Aya Megahed, Sahar E. Hamed, Eman A. El-Said

**Affiliations:** 1 Animal, Poultry and Fish Production Department, Faculty of Agriculture, Damietta University, Damietta, Egypt; 2 Animal Production Research Institute (APRI), Agriculture Research Center, Ministry of Agriculture, Dokki, Giza 12619 Egypt; 3 Department of Physiology, Faculty of Veterinary Medicine, Mansoura University, Mansoura, 35516 Egypt; 4 Department of Clinical Pathology, Faculty of Veterinary Medicine, Mansoura University, Mansoura, 35516 Egypt; 5 Department of Agricultural Biotechnology, Faculty of Agriculture, Damietta University, Damietta, Egypt

**Keywords:** Nanoemulsion orange essential oil, Heat stress, Growth performance, Oxidative stress, Inflammation, Liver and kidney function and structure

## Abstract

Heat stress (HS) adversely affects rabbit production, reducing growth performance, physiological responses, and farm profitability. Nanoemulsified orange essential oil (NOEO) is a natural antioxidant with anti-inflammatory properties that may mitigate oxidative stress and enhance cellular defense mechanisms. This study evaluated the effects of dietary NOEO on growth performance, blood biochemistry, antioxidant status, immune and inflammatory responses, and liver and kidney histomorphology in heat-stressed rabbits. Forty-eight six-week-old male New Zealand White rabbits (752.91 ± 17.40 g) were randomly assigned to four groups: a control group fed a basal diet and three treatment groups receiving the same diet supplemented with 100, 200, or 400 mg NOEO/kg feed. Heat-stressed rabbits supplemented with NOEO showed significant (*p* < 0.05) improvements in body weight, feed conversion ratio, and performance index. Blood hematology and serum levels of total protein, albumin, total cholesterol, triglycerides, low-density lipoprotein, and very low-density lipoprotein were also enhanced in all treated groups compared with the control. NOEO supplementation improved antioxidant defenses, as indicated by increased total antioxidant capacity and elevated activities of key antioxidant enzymes (glutathione peroxidase, catalase, and superoxide dismutase), particularly in the NOEO400 treated group (*p* < 0.05). Nitric oxide levels were significantly higher, while malondialdehyde concentrations were significantly lower in NOEO200 and NOEO400 groups, reflecting reduced oxidative stress (*p* < 0.05). Immune responses were strengthened, with higher serum IgM and IgA levels, accompanied by downregulation of pro-inflammatory cytokines IL-6 and IFN-γ (*p* < 0.05). Furthermore, liver and kidney function and tissue structure were improved in NOEO-supplemented rabbits. In conclusion, dietary NOEO, particularly at 400 mg/kg, enhanced growth performance, antioxidant status, immune competence, and hepato-renal health in heat-stressed rabbits, likely through improved cellular defense mechanisms that protect against oxidative and inflammatory damage.

## Introduction

Livestock production is highly susceptible to heat stress (HS), which represents one of the most serious challenges to animal health and productivity due to its detrimental effects on behavior, physiological, and immunological responses, fertility, growth performance, and meat quality (Abdelnour et al. 2020; Johnson 2018).

Rabbit production in Egypt has expanded in recent years to meet the growing demand for fresh meat and to support small-scale farmers (Abdel-Rahman and Ashour 2023). Rabbits maintain thermoneutrality within a temperature range of 15–25 °C, and exposure to higher temperatures induces HS (Oladimeji et al. 2022). HS occurs when an animal’s heat production exceeds its capacity for heat dissipation (Liang et al. 2022). Compared with other livestock species, rabbits are particularly vulnerable to HS because they lack functional sweat glands, limiting their ability to regulate body temperature and predisposing them to heat-related disorders (Oladimeji et al. 2022; Pasha et al. 2024). This vulnerability is further exacerbated by their high metabolic rate and elevated productive performance, which increase susceptibility to both environmental stressors (e.g., high ambient temperatures) and nutritional challenges (e.g., inadequate feed quantity or quality) (Ebeid et al. 2023). Consequently, HS impairs immune competence, increases susceptibility to pathogens and respiratory disorders, elevates heart rate, suppresses appetite and metabolic efficiency, and collectively reduce rabbit health and productivity (Liang et al. 2022; Nawaz et al. 2021).

Previous studies have shown that exposure to HS significantly impairs feed intake and feed conversion ratio in rabbits (Martignon et al. 2021; Szendrő et al. 2018). During the weaning phase, rabbits also experience substantial oxidative and inflammatory stress, which increases lipid peroxidation, compromises membrane integrity and intestinal barrier function, and ultimately leads to cellular damage (El-Deep et al. 2020). It is important to note, however, that protein denaturation is primarily associated with thermal insults rather than the physiological stress of weaning (Chen et al. 2022; Gugołek and Kowalska 2020). To mitigate the detrimental effects of HS on growth performance and meat quality, various nutritional and management strategies have been explored (Hashem et al. 2023; Mirghaed et al. 2020; Rahman et al. 2019).

The use of agro-industrial byproducts and natural product extracts containing bioactive ingredients has gained considerable attention in animal feed, both to reduce environmental impact and lower production costs (Attia et al. 2020). Furthermore, traditional poultry feedstuffs have sometimes contributed to lower growth performance in poultry (Abou-Kassem et al. 2020). In this context, the present study focuses on the emerging trend of phytogenic feed additives as alternatives, which can be categorized into four main subgroups: herbs, botanicals, essential oils (EOs), and oleoresins (Amer et al. 2019; Ogbuewu et al. 2020). These additives have been shown to positively influence growth performance, as well as immunological and antioxidant status in animals (Mahmoud et al. 2025).

Orange EO (OEO) has been applied in various agricultural, pharmaceutical, and biomedical contexts, either as extracts or EOs (Lee et al. 2018; Oprea et al. 2022). Dietary supplementation with OEO has been shown to enhance growth performance, immune function, and reproductive responses, owing to its antibacterial, antioxidant, and anti-inflammatory properties (Correddu et al. 2020; Rafiq et al. 2018). However, the use of EOs as dietary supplements is limited by their instability during storage, poor solubility, low bioavailability, and susceptibility to external factors such as UV light, pH fluctuations, and temperature changes, which restrict their broader application in the food industry and other sectors (Salanță and Cropotova 2022). Moreover, even low concentrations of EOs have been associated with organ toxicity, mucous membrane irritation, and respiratory issues (Mehdizadeh and Moghaddam 2018; Soni et al. 2023). Nevertheless, numerous studies have demonstrated that converting EOs into nanoparticles can enhance their biological and industrial properties, enable targeted delivery of bioactive compounds, and improve their suitability for incorporation into animal feed (El-Desoky et al. 2021, 2022; El-Raghi et al. 2023). Based on these considerations, the present study aimed to evaluate the effects of nanoemulsified OEO (NOEO) as a growth promoter on performance indices, blood hematology and biochemistry, antioxidant status, and immunological and inflammatory responses in heat-stressed rabbits.

## Materials and methods

### Ethical approval

The study was approved by the Research Ethics Committee, Faculty of Veterinary Medicine, Mansoura University, Egypt (registration code: MU-ACUC; VM.R.24.11.193). In cooperation with the Department of Physiology, Faculty of Veterinary Medicine, Mansoura University, Egypt and the Department of Animal, Poultry, and Fish Production, Faculty of Agriculture, Damietta University, Egypt, the current experiment was conducted at a privately-owned rabbit farm in Mansoura City, Dakahlia Governorate, Egypt. All procedures adhered to internationally recognized ethical standards.

### Experimental design

A total of forty-eight New Zealand White (NZW) male rabbits, weaned at six weeks of age, weighing 752.91 ± 17.40 g, were randomly allotted into 4 equal groups (12 rabbits/group). The control group was given the same diet without supplements, whereas the other three groups were given the same diet plus 100, 200, and 400 mg NOEO/kg diet.

### Preparation of NOEO

The Pure Life Company, based in Giza, Egypt, provided the OEO. A monolayer of NOEO, oil-in-water, was prepared according to the method outlined by Ullah et al. (2022). To summarize, OEO gradually dropped about 8 mL of water at a rate of 2 mL per minute to create nano-emulsions. Thereafter, the resultant nano-emulsion spent 30 min in an ultrasonic bath. Additionally, it was homogenized using a homogenizer-related ultrasonic probe to produce a nano-emulsion with OEO.

### Experimental animals, management, housing, and diets

The rabbits were kept separately in cages of 35 × 35 × 60 cm of galvanized wire for the duration of the experiment, which lasted from 6 to 14 weeks of age. Every rabbit was housed under the same management system for the duration of the study. Every day at 7 a.m., enough food was provided, and water was accessible through the cage nipple. The ration was formulated based on the NRC (1977) nutrient requirements for rabbits. The composition and chemical analysis of the experimental diets are shown in Table 1.

**Table 1:** Feed components and research diets’ chemical constituents (% DM basis)

Feed ingredients	%	Chemical analysis (%DM basis)	%
Soybean meal	18	Crude protein	17.5
Clover hay	36	Crude fiber	13.6
Yellow corn	19	Organic matter	88.4
Wheat bran	23	Dry matter	87.63
Molasses	2	Ether extract	2.2
NaCl	0.6	Nitrogen free extract	63.33
Premix*	0.6	Ash	6.95
Di-Ca-phosphate	0.4	Digestible energy (Kcal/Kg DM)	2563
DL-Methionine	0.4		

*The premix included a blend of minerals and vitamins per kilogram of diet, consisting of: 9000 IU of Vitamin A, 900 IU of Vitamin D3, 33 mg of Vitamin E, 2.3 mg of Vitamin K3, 0.7 mg of Vitamin B1, 3 mg of Vitamin B2, 0.003 mg of Vitamin B12, 0.1 mg of folic acid, 1.3 mg of iodine, 50 mg of manganese, 15.3 mg of copper, 65 mg of zinc, 50 mg of iron, 0.5 mg of cobalt, and 0.1 mg of selenium

### Meteorological parameters

The present study was carried out during the summer season in Egypt (June–August). Throughout the experimental period, the indoor ambient temperature (AT, °C) and relative humidity (RH, %) were measured daily at 9 a.m. and 1 p.m. using a thermohydrometer. The temperature-humidity index (THI) was then calculated based on this data using the specified equation outlined by Habeeb et al. (2018).

$$\:\mathbf{T}\mathbf{H}\mathbf{I}=\mathbf{t}-\left[\left(\boldsymbol{\mathrm{0.31-0.31}}\:\left(\frac{\mathbf{R}\mathbf{H}}{\boldsymbol{\mathrm{100}}}\right)\right)\:\times\:\:(\mathbf{t}\boldsymbol{\mathrm{-14.4}})\right]$$

Here, t represents the dry bulb temperature (°C), and RH denotes relative humidity. THI values were categorized as follows: less than 27.8 indicated no HS, 27.8 to 28.9 represented moderate HS, 29.0 to 30.0 indicated severe HS, and values above 30.0 signified very severe HS.

### Growth performance and feed utilization

Within the experimental period, lives body weights were recorded biweekly in the morning before providing additional feeds, simultaneously, daily feed intake was recorded by deducting weight of the remaining amounts of feed from the offered feed before adding the new ones at 7 a.m. The average daily gain (ADG), specific growth rate (SGR), performance index (PI) and feed conversion ratio (FCR) were estimated based on aforementioned data based on the following formulas.

$$\:\mathrm{A}\mathrm{D}\mathrm{G}=\:\left[\frac{(\mathrm{F}\mathrm{i}\mathrm{n}\mathrm{a}\mathrm{l}\:\mathrm{b}\mathrm{o}\mathrm{d}\mathrm{y}\:\mathrm{w}\mathrm{e}\mathrm{i}\mathrm{g}\mathrm{h}\mathrm{t}-\mathrm{I}\mathrm{n}\mathrm{i}\mathrm{t}\mathrm{i}\mathrm{a}\mathrm{l}\:\mathrm{b}\mathrm{o}\mathrm{d}\mathrm{y}\:\mathrm{w}\mathrm{e}\mathrm{i}\mathrm{g}\mathrm{h}\mathrm{t})}{\mathrm{D}\mathrm{u}\mathrm{r}\mathrm{a}\mathrm{t}\mathrm{i}\mathrm{o}\mathrm{n}}\right]$$  

$$\:\mathrm{S}\mathrm{G}\mathrm{R}=\:\left[\frac{(\mathrm{l}\mathrm{n}\:\mathrm{F}\mathrm{i}\mathrm{n}\mathrm{a}\mathrm{l}\:\mathrm{b}\mathrm{o}\mathrm{d}\mathrm{y}\:\mathrm{w}\mathrm{e}\mathrm{i}\mathrm{g}\mathrm{h}\mathrm{t}\hspace{0.17em}-\hspace{0.17em}\mathrm{l}\mathrm{n}\:\mathrm{I}\mathrm{n}\mathrm{i}\mathrm{t}\mathrm{i}\mathrm{a}\mathrm{l}\:\mathrm{b}\mathrm{o}\mathrm{d}\mathrm{y}\:\mathrm{w}\mathrm{e}\mathrm{i}\mathrm{g}\mathrm{h}\mathrm{t})}{\mathrm{D}\mathrm{u}\mathrm{r}\mathrm{a}\mathrm{t}\mathrm{i}\mathrm{o}\mathrm{n}}\right]$$  

$$\:\mathrm{P}\mathrm{I}=\:\left[\right(\mathrm{F}\mathrm{i}\mathrm{n}\mathrm{a}\mathrm{l}\:\mathrm{b}\mathrm{o}\mathrm{d}\mathrm{y}\:\mathrm{w}\mathrm{e}\mathrm{i}\mathrm{g}\mathrm{h}\mathrm{t},\:\mathrm{K}\mathrm{g}\:\times\:\:100)/\mathrm{F}\mathrm{C}\mathrm{R}]$$

$$\:\mathrm{F}\mathrm{C}\mathrm{R}=\:(\mathrm{g}\:\mathrm{f}\mathrm{e}\mathrm{e}\mathrm{d}/\mathrm{g}\:\mathrm{g}\mathrm{a}\mathrm{i}\mathrm{n})$$

### Blood hematology

At the end of the experimental period, blood samples were collected from the ear vein of five rabbits after applying a local anesthetic cream to the site. The samples were drawn into sterile tubes and divided into two subsamples. The first subsample was analyzed for hematological parameters using an automated hematology analyzer (Hospitex Hema Screen 18, Sesto Fiorentino, Italy), following the methods described by Schalm et al. (1975). The second subsample was left to clot, then centrifuged at 3500 rpm for 15 min. The resulting serum was separated and stored at -20 °C for subsequent analysis.

### Blood profile

The concentrations of serum total proteins (TP), albumin (Alb), globulin (Glb), albumin/globulin ratio (A/G ratio), aspartate aminotransferase (AST), alanine aminotransferase (ALT), glutamyl transferase (GGT), urea, creatinine, triglycerides (TG), total cholesterol (TC), high-density lipoprotein (HDL), low-density lipoprotein (LDL), and very low-density lipoprotein (VLDL) were spectrophotometrically estimated using commercial kits obtained from Bio-diagnostic Company (Giza, Egypt) following the instructions of the manufacturer.

Redox status, including total antioxidant capacity (TAC), glutathione peroxidase (GSH-Px), catalase (CAT), superoxide dismutase (SOD), and malondialdehyde (MDA), was assayed using specialized quantitative sandwich ELISA kits. The level of serum nitric oxide (NO) was determined using the methodology outlined by Rajaraman et al. (1998). Regarding the inflammatory estimation, the levels of serum interleukin-6 (IL-6) and interferon-γ (IFN-γ) were quantitated using ELISA kits assay (MyBioSource, San Diego, CA, USA). Furthermore, immunoglobulin M and A (IgM and IgA) levels were determined by ELISA kits.

### Histological investigation

At the end of the experiment, liver and kidney samples were obtained from slaughtered rabbits for the purpose of histological examination. The collected samples were fixed in a 10% neutral formalin buffer to preserve their structure and composition. To prepare the specimens, a series of steps were performed. First, the specimens were dehydrated using increasing concentrations of ethyl alcohol. Subsequently, they were cleared in two changes of xylol, a substance commonly used to remove alcohol from tissue samples. The dehydrated and cleared liver and kidney specimens were then embedded in paraffin blocks to facilitate the cutting process. Thin sections, approximately 4 μm thick, were sliced from the paraffin blocks using a microtome (specifically, the Leica RM 2155 model). To visualize the cellular structures and tissue components, the liver and kidney sections were subjected to hematoxylin and eosin (H&E) staining. This staining method is commonly employed in histology to distinguish cellular nuclei (hematoxylin) from cytoplasmic components (eosin), facilitating meticulous analysis under light microscopy. The stained sections were observed and analyzed using an Olympus CX31 microscope. To document the findings, representative photomicrographs were captured using a digital camera microscope (Leica DM 500, Leica EC3, Leica).

### Analytical statistics

Microsoft Excel was used to handle and organize the data (Microsoft Corporation, Redmond, WA, USA). To evaluate normality, a Shapiro-Wilk test was performed as outlined by Razali and Wah (2011). Statistical assessment was conducted using One-Way ANOVA (PROC ANOVA; SAS, 2012) to evaluate the impact of NOEO treatments on growth performance, feed utilization, and hemato-biochemical parameters, based on the following model: Yij = µ + TRTi + eij.

In case of, Yij = Observations, µ = Overall mean, TRT = refers of i^th^ antioxidant material (i, 1 to 4), eij = random error. Results were expressed as means ± SE. Pairwise comparisons across means were expressed using Tukey’s test when a significant effect was found. A p-value of less than 0.05 was used to indicate statistical significance across means.

## Results

### Characterization of NOEO formation

**Fig. 1:**
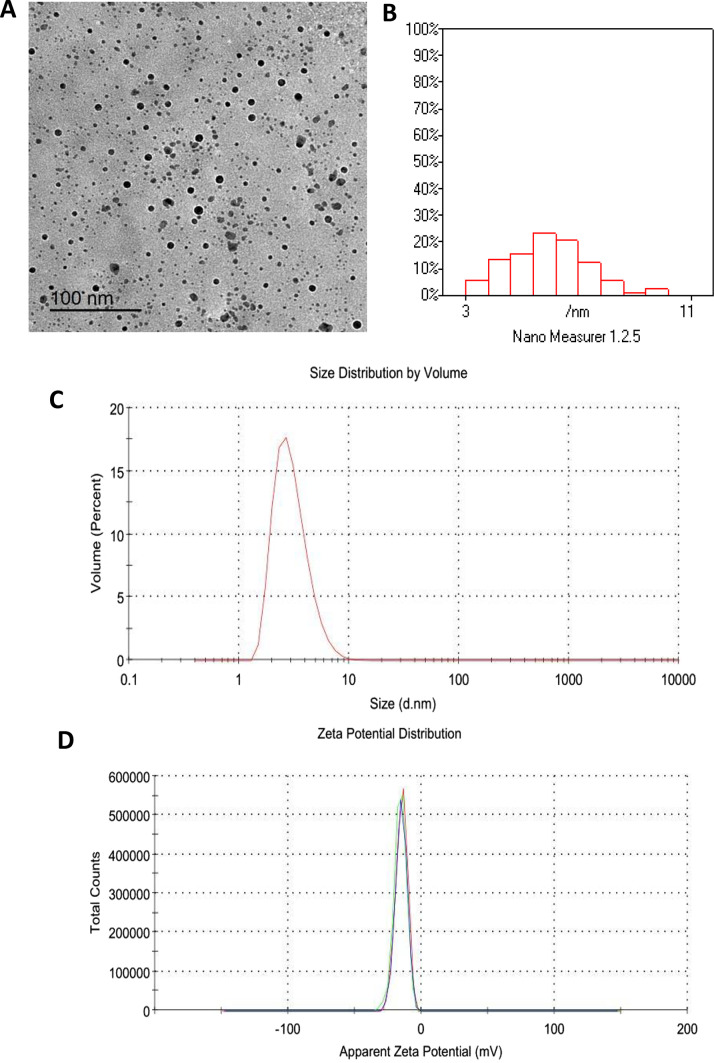
**A**) TEM (JEOL-JEM 2100) image showing NOEO particles with a nearly spherical morphology and minimal aggregation. **B**) Frequency distribution of nanoparticle sizes across different ranges, including descriptive statistics such as mean size and frequency percentages. **C**) Zeta size distribution by volume. **D**) Zeta potential distribution. The measured values of Z-average size, PDI, and ZP of NOEO are also presented

Figure 1A-D illustrates the characterization of NOEO. Transmission electron microscopy (TEM) revealed that NOEO particles are nearly spherical with minimal aggregation (Fig. 1A). Particle size analysis showed a range of 3–11 nm (Fig. 1B). Dynamic light scattering (DLS) determined an average particle size of 5 nm with a polydispersity index (PDI) of 0.26 (Fig. 1C). Zeta potential (ZP) analysis indicated a negative surface charge of -15.1 mV (Fig. 1D).

### Meteorological parameters

Table 2 displays the climatic parameter results for the experimental period. The average AT, RH, and calculated THI were estimated to be 34.01 ± 0.17 °C, 66.12 ± 0.37%, and 31.95 ± 0.13, respectively. The newly weaned rabbits had severe HS, according to the THI values.

**Table 2:** The average temperature, relative humidity, and temperature-humidity index for the course of the experiment

Parameters	July	August	Overall
AT (°C)	33.78 ± 0.15	34.26 ± 0.14	34.01 ± 0.12
RH (%)	65.30 ± 0.42	66.94 ± 0.18	66.12 ± 0.37
THI	31.69 ± 0.14	32.20 ± 0.13	31.95 ± 0.13

AT: ambient temperature; RH: relative humidity; THI: temperature–humidity index

### Growth performance and feed utilization

Results in Table 3 show the effect of dietary supplementation with NOEO on growth performance and feed utilization in heat-stressed growing rabbits. The values of FBW, WG, ADG, and PI were improved significantly by treatment (*p* < 0.001). The NOEO400-treated group showed the highest growth performance compared to the control and the other treated groups (*p* < 0.05); non-significant differences were detected between the NOEO100- and NOEO200-treated groups (*p* > 0.05). Feed conversion ratio decreased significantly by the dietary treatment (*p* < 0.0001), minimizing in the NOEO400-treated group. However, the treatment did not significantly affect SGR and FI (*p* = 0.7502).

**Table 3:** Growth performance and feed utilization of heat-stressed rabbits fed diets supplemented with different doses from NOEO

Items	NOEO0	NOEO100	NOEO200	NOEO400	SEM	*p*-value
IBW (g)	755.92	749.83	756.17	749.75	15.115	0.6856
FBW (g)	2023.42^**c**^	2173.92^**b**^	2204.33^**b**^	2240.83^**a**^	11.474	< 0.0001
WG (g)	1267.50^**c**^	1424.08^**b**^	1448.17^**b**^	1491.08^**a**^	9.976	< 0.0001
ADG (g)	22.63^**c**^	25.43^**b**^	25.86^**b**^	26.63^**a**^	0.178	< 0.0001
SGR (%, d)	1.76	1.90	1.91	1.96	0.011	< 0.0001
FI (g)	110.08	107.17	106.92	108.00	2.259	0.7502
FCR (g feed/g gain)	4.86^**a**^	4.21^**b**^	4.14^**b**^	4.06^**b**^	0.088	< 0.0001
PI (%)	41.67^**c**^	51.82^**b**^	53.66^**ab**^	55.66^**a**^	1.252	< 0.0001

NOEO0, NOEO100, NOEO200 and NOEO400 = 0, 100, 200 and 400 g NOEO/kg diet, respectively. IBW, initial body weight; FBW, final body weight; WG, weight gain; DWG, daily weight gain; SGR, specific growth rate; FI, feed intake; FCR, Feed conversion ratio; PI, performance index; ^a, b,c^ Means in the same row with different superscript letter following them are significantly different (*p* < 0.05)

### Blood hematology

Table 4 shows the impact of NOEO dietary supplementation on hematological markers. Hemoglobin concentration was significantly improved in all treated groups compared to the control (*p* < 0.05). Erythrocyte and leucocyte counts were significantly higher in all treated groups compared to the control (*p* < 0.05), but both the NOEO200- and NOEO400-treated groups showed more significant improvement in these parameters than the NOEO100-treated group (*p* < 0.05). The number of platelets was markedly improved in the NOEO200- and NOEO400-treated groups compared to the NOEO100 and control groups (*p* < 0.05). Meanwhile, non-significant differences were observed between the values of HCT, PCV, MCV, MCH, MCHC, LYM, MONO, and NEUT as a response to the dietary treatment (*p* > 0.05).

**Table 4:** Hematological attributes of rabbits under heat stress fed diets enhanced with different doses from NOEO

Items	NOEO0	NOEO100	NOEO200	NOEO400	SEM	*p*-value
HGB (g/dl)	10.073^**b**^	11.68^**a**^	12.13^**a**^	11.98^**a**^	0.37	0.0155
RBCs (10^6^/µl)	4.60^**c**^	5.18^**b**^	6.40^**a**^	6.20^**a**^	0.12	< 0.0001
WBCs (10^3^/mm^3^)	5.39^**c**^	6.38^**b**^	6.68^**ab**^	7.31^**a**^	0.25	0.0067
PLT (10^3^/mm^3^)	282.70^**b**^	301.88^**b**^	364.52^**a**^	373.99^**a**^	12.56	0.0019
PCV (%)	31.5	33.1	32.3	34.4	2.48	0.9137
HCT (%)	26.72	28.07	27.28	26.40	2.48	0.9657
MCV (fl.)	56.97	59.10	54.18	57.36	1.89	0.3824
MCH (pg)	22.73	21.96	21.75	19.77	3.13	0.9185
MCHC (g/dl)	41.79	42.89	41.813	43.26	1.43	0.8444
LYM (%)	38.51	36.72	37.46	37.91	0.85	0.5375
MONO (%)	8.55	8.22	8.61	8.46	0.33	0.8590
NEUT (%)	49.47	50.86	49.04	50.27	0.58	0.2001

NOEO0, NOEO100, NOEO200 and NOEO400 = 0, 100, 200 and 400 g NOEO/kg diet, respectively. HGB, hemoglobin; RBCs, red blood cells; WBCs, white blood cells; PLT, platelet count; PCV, Packed cell volume; HCT, hematocrit; MCV, mean corpuscular volume; MCH, mean corpuscular hemoglobin; MCHC, mean corpuscular hemoglobin concentration; LYM, lymphocytes; MONO, monocytes; NEUT, Neutrophils; ^**a, b,c**^ Means in the same row with different superscript letter following them are significantly different (*p* < 0.05)

### Blood profile

The concentrations of blood TP and Alb were significantly higher in the NOEO400- and NOEO200-treated groups than in the NOEO100 and control groups (*p* < 0.05). Meanwhile, non-significant differences were observed between the values of Glb and A/G as a response to the dietary treatment (*p* > 0.05). The activities of AST, ALT, and GGT were significantly reduced in all treated groups compared to the control (*p* < 0.05), with the lowest values observed in the NOEO400 group. Furthermore, there are significant improvements in kidney functions; serum urea and creatinine were significantly lower in all treated groups than in the control (*p* < 0.05). The concentrations of TG, TC, LDL, and VLDL were significantly decreased in comparison to the control in all treatment groups (*p* < 0.05), but HDL showed a non-significant response to the dietary treatment (*p* > 0.05; Table 5).

**Table 5:** Blood profile of heat-stressed rabbits fed diets supplemented with different doses from NOEO)

Items	NOEO0	NOEO100	NOEO200	NOEO400	SEM	*p*-value
TP (g/dL)	6.07^**c**^	6.39^**bc**^	7.13^**a**^	6.88^**ab**^	0.17	0.0118
Alb (g/dL)	3.25^**c**^	3.44^**bc**^	3.76^**ab**^	3.82^**a**^	0.11	0.0174
Glb (g/dL)	2.82	2.95	3.36	3.06	0.17	0.2199
A/G ratio	1.15	1.18	1.13	1.25	0.08	0.7306
Liver function						
AST (IU)	38.91^**a**^	33.26^**b**^	35.23^**ab**^	30.38^**b**^	1.60	0.0310
ALT(IU)	55.34^**a**^	49.95^**ab**^	43.80^**b**^	43.70^**b**^	2.30	0.0201
GGT (mg/dL)	51.74^**a**^	43.71^**b**^	38.12^**bc**^	32.22^**c**^	1.99	0.0007
Kidney function						
Urea (mg/dL)	36.01^**a**^	29.06^**ab**^	26.00^**b**^	26.10^**b**^	2.15	0.0348
Creatinine (mg/dL)	1.51^**a**^	1.21^**b**^	1.16^**b**^	1.25^**b**^	0.07	0.0477
Lipid profile						
TG (mg/dL)	95.89^**a**^	88.70^**b**^	81.24^**b**^	72.76^**c**^	2.68	0.0015
TC (mg/dL)	107.20^**a**^	86.99^**c**^	91.11^**bc**^	89.00^**c**^	4.29	0.0455
HDL (mg/dL)	43.00	47.03	46.57	52.99	3.81	0.0877
LDL (mg/dL)	45.02^**a**^	22.21^**b**^	28.28^**b**^	21.45^**b**^	3.63	0.0062
VLDL (mg/dL)	19.17^**a**^	17.74^**b**^	16.24^**b**^	14.55^**c**^	0.44	0.0015

NOEO0, NOEO100, NOEO200 and NOEO400 = 0, 100, 200 and 400 g NOEO/kg diet, respectively. TP, total protein; Alb, albumin; Glb, globulin; A/G ratio, albumin/globulin ratio; AST, aspartate aminotransferase; ALT, alanine aminotransferase; GGT, glutamyl transferase; TG, triglycerides; TC, total cholesterol; HDL, high-density lipoprotein; LDL, low‐density lipoprotein; VLDL, very low‐density lipoprotein; ^a, b,c^ Means in the same row with different superscript letter following them are significantly different (*p* < 0.05)

### Redox status

Results in Table 6 demonstrate a significant improvement in redox balance due to the dietary treatment; TAC was significantly higher in all treated groups than the control group (*p* < 0.05). Additionally, the activities of key antioxidant enzymes, including GSH-Px, CAT, and SOD, were significantly higher in the NOEO400-treated group than the other studied groups (*p* < 0.05). The concentration of NO was significantly increased in the NOEO400-treated group compared to the other studied groups (*p* < 0.05). Regarding lipid peroxidase, the dietary treatment had a significant impact on MDA levels (*p* = 0.0183), being significantly lower in both the NOEO400- and NOEO200-treated groups than the control group (*p* < 0.05).

**Table 6:** Redox status of heat-stressed rabbits fed diets supplemented with different doses from NOEO

Items	NOEO0	NOEO100	NOEO200	NOEO400	SEM	*p*-value
TAC (mmol/L)	0.66^**c**^	0.82^**bc**^	1.31^**a**^	1.03^**b**^	0.06	0.0008
GSH-Px (U/L)	0.88^**c**^	1.18^**b, c**^	1.69^**ab**^	1.90^**a**^	0.16	0.0081
CAT (U/L)	369.67^**b**^	422.68^**b**^	441.66^**b**^	621.04^**a**^	36.67	0.0064
SOD (U/L)	22.78^**b**^	28.77^**b**^	27.42^**b**^	38.48^**a**^	2.65	0.0179
NO (Umol/L)	23.02^**b**^	28.89^**ab**^	32.76^**ab**^	37.28^**a**^	3.16	0.0437
MDA (nmol/L)	2.57^**a**^	2.35^**ab**^	2.06^**bc**^	1.74^**c**^	0.14	0.0183

NOEO0, NOEO100, NOEO200 and NOEO400 = 0, 100, 200 and 400 g NOEO /kg diet, respectively. TAC, total antioxidant capacity; GSH-Px, glutathione peroxidase; CAT, catalase; SOD, superoxide dismutase; NO, nitric oxide; MDA, malondialdehyde; ^a, b,c^ Means in the same row with different superscript letter following them are significantly different (*p* < 0.05)

### Immunity status and inflammatory response

The effects of dietary supplementation of NOEO on immuneglobulins IgM and IgA are presented in Table 7; the dietary treatment exhibited a significant increase in the aforementioned two cellular immunities, maximizing in the NOEO400 and NOEO200 groups compared to the NOEO100 and control groups (*p* < 0.05). With respect to pro-inflammatory cytokines, the level of IL-6 was significantly decreased in the NOEO400 and NOEO200 groups compared to the NOEO100 and control groups (*p* < 0.05), while the IFN-γ level was significantly decreased in all treated groups compared to the control (*p* < 0.05).

**Table 7:** Immunity status and inflammatory response of heat-stressed rabbits fed diets supplemented with different doses from NOEO

Items	NOEO0	NOEO100	NOEO200	NOEO400	SEM	*p*-value
Immunity						
IgM (mg/dL)	118.51^**c**^	128.65^**bc**^	144.07^**ab**^	156.58^**a**^	5.17	0.0038
IgA (mg/dL)	341.29^**b**^	371.13^**ab**^	443.38^**a**^	452.05^**a**^	27.16	0.0119
Pro-Inflammatory cytokines						
IL-6 (pg/mL)	185.52^**a**^	173.70^**ab**^	154.69^**b**^	121.94^**c**^	5.98	0.0003
IFN-γ (pg/mL)	37.53^**a**^	22.38^**b**^	21.17^**b**^	23.87^**b**^	2.06	0.0017

NOEO0, NOEO100, NOEO200 and NOEO400 = 0, 100, 200 and 400 g NOEO/kg diet, respectively. IgM, immunoglobulin M; IgA, immunoglobulin A; IL-6, interleukin-6; IFN-γ, interferon-γ; ^a, b,c^ Means in the same row with different superscript letter following them are significantly different (*p* < 0.05)

### Histological investigation

**Fig. 2:**
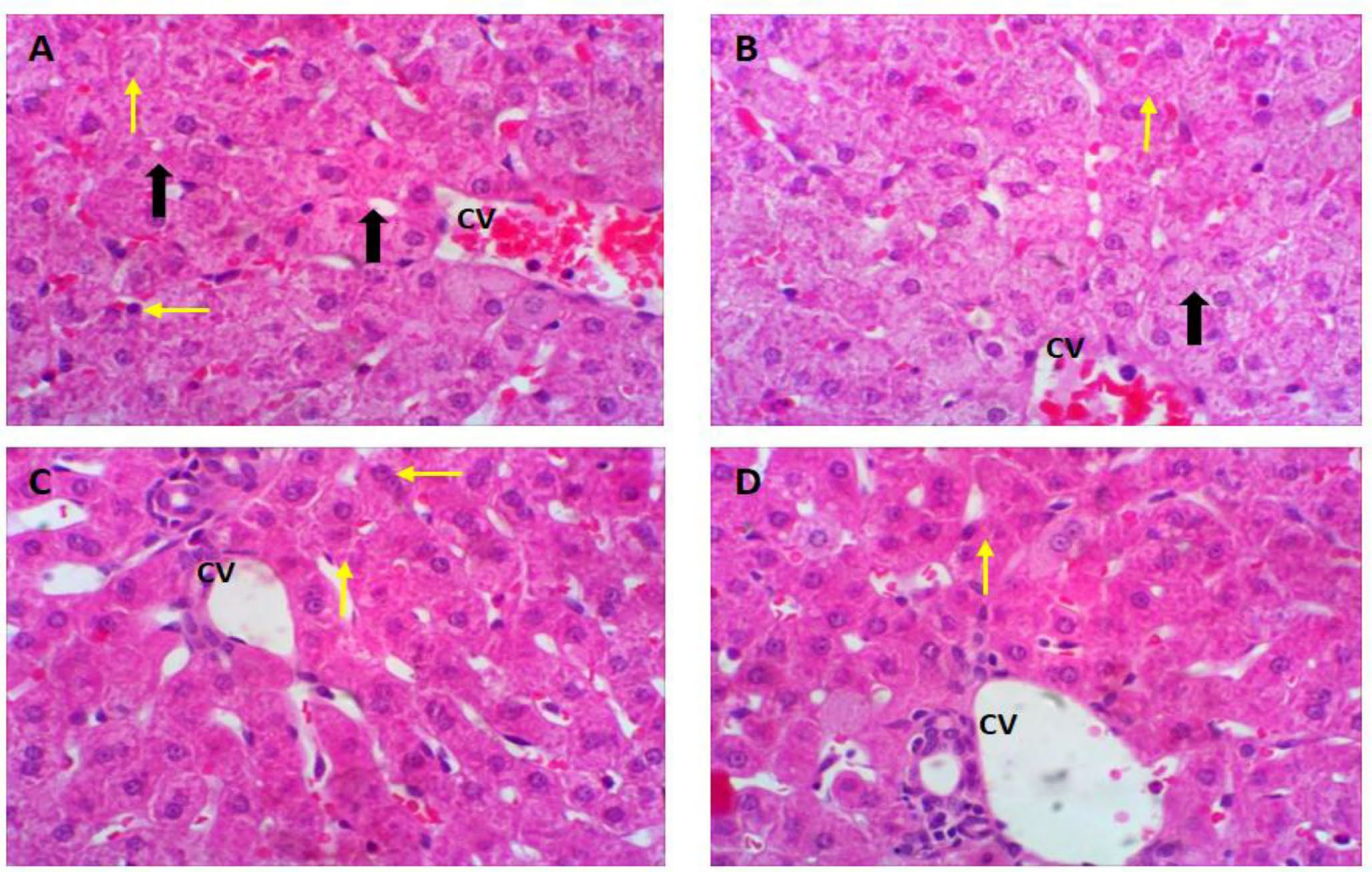
Representative photomicrographs of H&E stained liver tissue of growing rabbits in different treated groups (**A**; NOEO0, **B**; NOEO100, **C**; NOEO200, and **D**; NOEO400) (**A**) Liver tissue from rabbits under HS showing several pathological changes were observed, including hepatocellular necrosis (yellow arrow), multiple cytoplasmic vacuolated (thick arrow), blood infiltration and dilatation of the hepatic sinuses, and congestion of the hepatic central vein (CV). (**B**) Liver tissue of rabbits that was treated by 100 mg NOEO showing mild to moderate alterations in the histopathological changes, along with the presence of small cytoplasmic vacuolated and mild congested CV. (**C** & **D**) Liver tissues of rabbits that were treated by 200 or 400 mg NOEO showing the liver tissue appeared normal, with hepatocytes (yellow arrow) arranged around the hepatic CV. The hepatic lobules and nuclei of hepatocytes were normal and intact. H&E staining, magnification = 400x

Representative photomicrographs of rabbit’s liver in different treated groups are depicted in Fig. 2A-D. The NOEO0 group of rabbits exposed to heat stress showed several pathological changes, including hepatocellular necrosis, multiple cytoplasmic vacuoles, blood infiltration and dilatation of the hepatic sinusoids, and congestion of the hepatic vein (Fig. 2A). The NOEO100-treated group showed mild to moderate alterations in the histopathological changes, along with the presence of small cytoplasmic gaps and a mildly congested central vein (Fig. 2B). NOEO200- and NOEO400-treated groups showed normal liver tissue, with hepatocytes arranged around the hepatic vein (Fig. 2C&D).

**Fig. 3:**
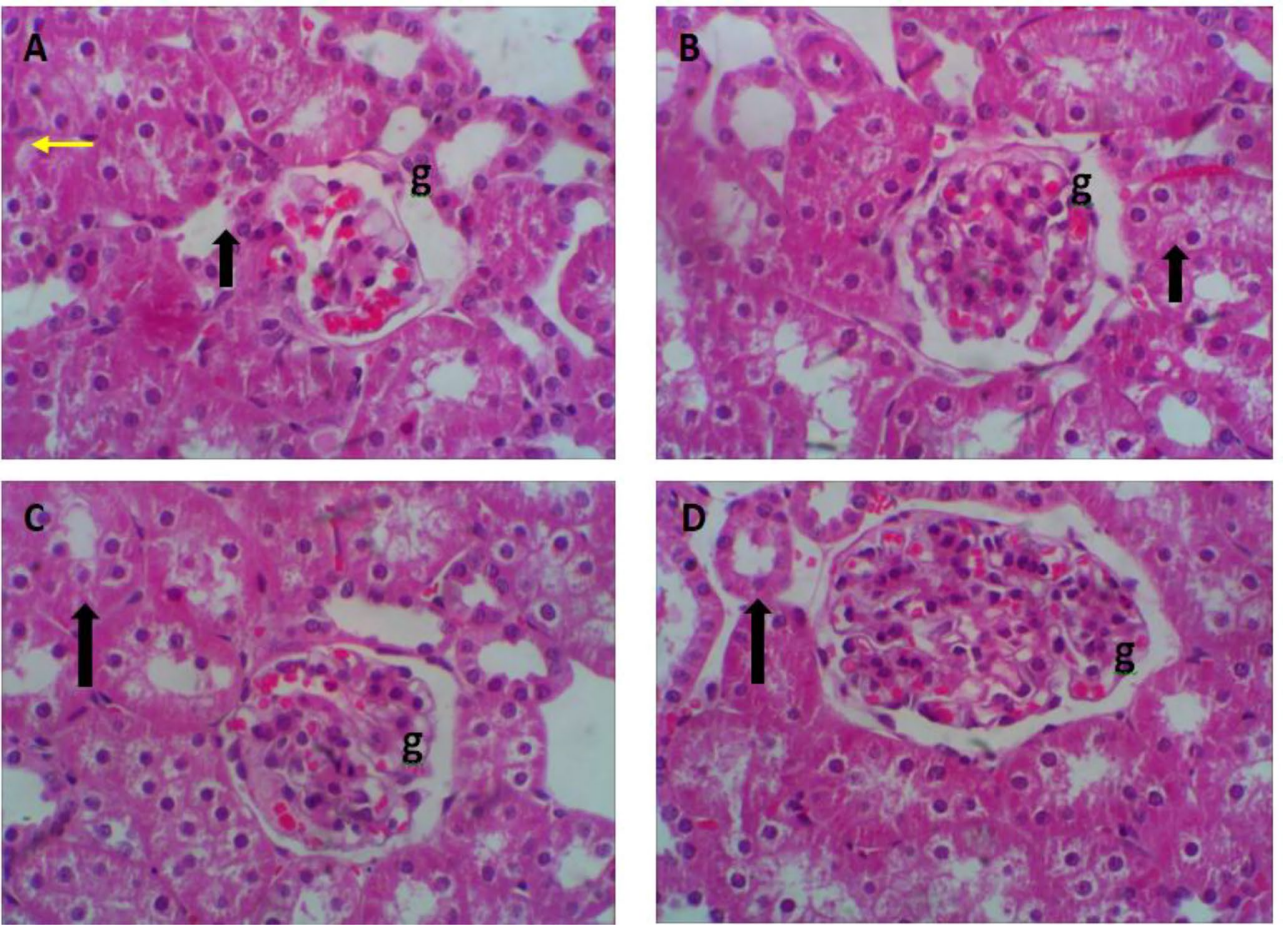
Representative photomicrographs of H&E stained kidney tissue of growing rabbits in different treated groups (**A**; NOEO0, **B**; NOEO100, **C**; NOEO200, and **D**; NOEO400) **(A)** Kidney tissue from rabbits under HS exhibited several pathological changes. Cytoplasmic vacuolations were observed in most of the tubules (black arrows), while others displayed epithelial desquamation. Additionally, many cells with darkly stained nuclei were visible (yellow arrows). Glomeruli (g) appeared shrunken or atrophied with wide Bowman´s space. Some congestion was apparent in the glomerulus (g). (**B**) Kidney tissue from rabbits treated with 100 mg of NOEO showed mild to moderate histopathological alterations. (**C** & **D**) Kidney tissues from rabbits treated with 200 mg or 400 mg of NOEO exhibited a normal glomerulus (g) surrounded by visceral and parietal layers of Bowman’s capsule, separated by Bowman’s space, and tubules were also observed. H&E staining, magnification = 400x

With regard to the histological investigation of the kidney, representative photomicrographs of rabbit kidneys in different treated groups are illustrated in Fig. 3A-D. The NOEO0 group without supplementation exhibited several pathological changes, including cytoplasmic vacuolations in most of the tubules. Additionally, many cells with darkly stained nuclei were visible. Moreover, glomeruli appeared shrunken or atrophied with wide Bowman’s space, and some congestion appeared in the glomerulus (Fig. 3A). The NOEO100-treated group showed mild to moderate histopathological alterations (Fig. 3B). NOEO200 and NOEO400 treated groups exhibited a normal glomerulus surrounded by visceral and parietal layers of Bowman’s capsule, separated by Bowman’s space, and tubules were also observed (Fig. 3C&D).

## Discussion

Rabbits have sweat glands with limited functionality, making them unable to efficiently eliminate excess body heat and, consequently, highly susceptible to HS. HS induces several physiological changes that reduce performance indices and impair immune responses (Oladimeji et al. 2022; Pasha et al. 2024; Shalaby et al. 2025). Therefore, this study was designed as a trial to mitigate the detrimental effects of HS through dietary supplementation with NOEO. The nano-emulsion form was employed to enhance absorption across the intestinal mucus layer, thereby improving the bioavailability and permeability of NOEO’s active components (Guo et al. 2021).

The present study demonstrated that dietary supplementation with NOEO markedly alleviated the adverse effects of HS in growing rabbits. Specifically, NOEO supplementation at 400 mg/kg significantly improved growth performance, feed utilization, and hematological parameters, while also enhancing liver and kidney function. Furthermore, NOEO increased TAC and the activities of GSH-Px, CAT, and SOD, accompanied by a reduction in MDA levels, indicating a restoration of redox balance. The treatment also improved immune status, as evidenced by elevated IgM and IgA levels and downregulation of IL-6 and IFN-γ. Histological analyses revealed that NOEO at both 200 and 400 mg/kg restored normal hepatic and renal architecture, confirming its protective efficacy against HS-induced cellular injury.

The temperature-humidity index, a biomarker for HS in animals (Li et al. 2023), reflects circulatory and metabolic responses to excessive heat by increasing respiratory evaporation and respiration rate (Rashamol et al. 2020). HS has a substantial negative impact on rabbit meat yield and quality, feed consumption, growth performance, antioxidant enzyme activity, fertility, and reproductive function (El-Ratel et al. 2023). Consistent with these findings, the present study recorded THI values ranging from 31.69 to < 32.50 °C, confirming severe HS conditions that adversely affected FBW, FCR, and PI in growing rabbits.

Nonetheless, rabbits supplemented with NOEO exhibited improved thermotolerance under high ambient temperatures, showing significant increases in FBW, FCR, and PI, particularly at the higher inclusion level (400 mg NOEO/kg diet). These findings are consistent with previous reports demonstrating that natural antioxidants can effectively reduce rectal temperature and enhance growth performance in heat-stressed rabbits (Abdel-Wareth et al. 2018; Elmorsy et al. 2025; Liang et al. 2022). Furthermore, the phytochemical components of NOEO have been shown to improve rabbits’ resistance to HS, enhance growth performance and carcass yield, and reduce abdominal fat, as reported by Abd El-Hamid et al. (2023) and Badawy et al. (2025), providing a rationale for the elevated PI observed in NOEO-supplemented rabbits in the present study.

Hematological parameters serve as sensitive indicators of physiological and immunological status in rabbits, reflecting their adaptability to environmental and nutritional stressors, including HS (Saghir et al. 2023). In the present study, dietary supplementation with NOEO significantly enhanced hemoglobin concentration, erythrocyte, platelet, and leukocyte counts, particularly at higher inclusion levels (200 and 400 mg/kg diet), indicating improved oxygen-carrying capacity, immune competence, and hematopoietic function. These findings suggest that NOEO may mitigate the hematological suppression typically induced by HS through its bioactive components and antioxidant capacity (El-Gindy et al. 2023; Valdivieso-Ugarte et al. 2019), which help preserve erythrocyte membrane integrity and support bone marrow activity (Chircov et al. 2021). In contrast, exposure to elevated temperatures is known to depress hematological indices by impairing erythropoiesis and inducing oxidative damage to blood cells, predisposing animals to immunosuppression and infection (Mylostyvyi 2025). Therefore, the improved hematological profile observed in NOEO-supplemented groups highlights its potential role to counteract HS-induced physiological disturbances and maintain hematological stability under thermal stress conditions.

Additionally, blood biochemical markers reflect the physiological responses of rabbits to both internal and external stressors. In the present study, serum TP and Alb levels were significantly elevated in NOEO-supplemented groups, despite the fact that serum TP typically declines under HS (Liang et al. 2022). This increase may be attributed to the hepatoprotective and antioxidant properties of NOEO, which can enhance liver function and promote protein synthesis (El-Said et al. 2025; Ismail et al. 2023), thereby indicating an improved nutritional and physiological status in the treated rabbits. Crucially, the measured values stayed within the physiological reference range documented for healthy rabbits, indicating that the rise is not a pathogenic change but rather an advantageous adaptive response (Kurtz and Travlos 2017; Oglesbee 2020; Sherif et al. 2018). Regarding the lipid profile, previous studies have reported that OEO exhibits a hypocholesterolemic effect (Caceda-Gallardo et al. 2020). Similarly, in the present study, dietary supplementation with NOEO in heat-stressed rabbits resulted in a significant reduction in serum TG, TC, LDL, and VLDL levels. These effects may be attributed to the ability of EO constituents to regulate cholesterol biosynthesis and lipid metabolism (Feng et al. 2020; Ji et al. 2019).

Heat stress is well known to induce excessive generation of reactive oxygen species (ROS), which can overwhelm the endogenous antioxidant defense system and cause oxidative damage to cellular lipids, proteins, and DNA (Aryal et al. 2025; Khan et al. 2024). This oxidative imbalance disrupts mitochondrial function, impairs metabolic processes, and promotes inflammation, ultimately compromising growth and overall health in rabbits (Ebeid et al. 2023). Therefore, rapid neutralization of ROS by antioxidant systems is crucial to prevent oxidative injury and maintain physiological homeostasis under HS conditions (Elmorsy et al. 2025; Rao et al. 2025).

Consistent with these mechanisms, the present study demonstrated that dietary supplementation with NOEO at 200 and 400 mg/kg diet significantly enhanced antioxidant enzyme activities, as evidenced by increased levels of TAC, GSH-Px, CAT, and SOD, alongside reduced MDA concentrations compared with the control group. These results confirm that NOEO effectively mitigates oxidative stress in heat-stressed rabbits. GSH-Px, CAT, and SOD serve as the primary enzymatic antioxidants forming the first line of defense against ROS (Embaby et al. 2025b; Eldesoqui et al. 2025). By scavenging free radicals and reducing lipid peroxidation, these enzymes protect cellular structures, maintain redox homeostasis, and preserve cellular integrity under stress conditions (Eldesoqui et al. 2024). These findings are consistent with the well-known biological activity of phenolic and antioxidant compounds in OEO, which protect cells against ROS-mediated damage, including superoxide, peroxyl radicals, hydroxyl radicals, and peroxynitrite, by enhancing serum antioxidant enzyme activity (Elazab et al. 2022; Serreli and Deiana 2020). In this context, NOEO exhibits strong antioxidant potential due to its bioactive constituents, such as limonene and linalool, and is considered non-toxic, making it suitable for long-term dietary supplementation (Al-Maqtari et al. 2022).

The present data showed that NO concentration was significantly increased in NOEO-treated groups compared to the control group. NO acts as an antioxidant and markedly elevates anti-inflammatory and vasodilatory responses (Papi et al. 2019). In response to increases in body temperature, NO promotes vasodilation of arteries and major blood vessels, thereby helping to regulate body temperature and alleviate heat stress in rabbits (Jaswal et al. 2025). Additionally, NO functions as an antioxidant by neutralizing free radicals and can modulate the release of numerous inflammatory mediators from various immune cells (Embaby et al. 2025a), highlighting its importance in improving heat tolerance in NOEO-treated rabbits.

Beyond its antioxidative role, NOEO supplementation also enhanced immune and inflammatory responses in heat-stressed rabbits. HS is known to elevate pro-inflammatory cytokines, such as IL-6 and IFN-γ, which are crucial for immune regulation but, when excessively produced, contribute to oxidative and inflammatory damage (Rosi et al. 2021; Sheiha et al. 2020). Consistent with these findings, the present study observed increased IL-6 and IFN-γ levels in heat-stressed rabbits, reflecting heightened intestinal permeability and susceptibility to infections (Abd El-Hack et al. 2020). Consequently, the reduced productive performance observed in heat-stressed rabbits may be linked to immune suppression and increased pathogen load.

Interestingly, NOEO supplementation, particularly at 400 mg/kg, significantly increased serum IgM and IgA concentrations while reducing IL-6 and IFN-γ levels, indicating improvements in both humoral immunity and inflammatory regulation. IgM and IgA are key immunoglobulins responsible for systemic and mucosal defense, respectively; their elevation suggests enhanced intestinal barrier protection and stronger immune resilience (Chen et al. 2020). The immunomodulatory effects of NOEO likely arise from the combined antioxidant, anti-inflammatory, antimicrobial, and immunostimulatory properties of its bioactive compounds (Al-Harrasi et al. 2022; Aziz et al. 2018; Sandner et al. 2020). By reducing oxidative stress and suppressing pro-inflammatory cytokine signaling, NOEO helps preserve immune cell function and prevent chronic inflammation. Collectively, these findings demonstrate that NOEO confers dual protective benefits under heat stress by reinforcing antioxidant defenses and modulating immune and inflammatory pathways. This integrated protection enhances physiological stability, immune competence, and overall performance in rabbits exposed to thermal stress.

Many metabolic systems are disrupted in response to HS, with declines in liver and kidney function serving as key indicators of this metabolic disturbance (Li et al. 2021). The liver, as the main metabolic organ, relies on Kupffer cells to act as an effective filtration system, preventing particulate matter or bacteria from the digestive system from entering systemic circulation via portal blood (El-Raghi et al. 2023). HS-induced liver disorders may result in elevated liver enzymes, intrahepatic cholestasis, low platelet syndrome, and increased hepatocyte damage (Wang et al. 2022). Additionally, muscle tissue breakdown under HS can contribute to kidney dysfunction. Therefore, maintaining liver and kidney function and structural integrity is critical to mitigate the adverse physiological effects of HS.

The present findings demonstrated that dietary supplementation with NOEO had a significant positive effect on liver enzyme activity, including ALT, AST, and GGT, which serve as key indicators of liver function. Low serum GGT levels in NOEO-treated groups may reflect improved liver health, efficient bile duct secretion, and reduced leakage of liver enzymes into the bloodstream (Abdelnour et al. 2019). A positive statistical correlation between liver enzyme activity and serum protein levels further indicates enhanced liver function in NOEO-supplemented groups compared to controls. These results support the role of OEO in enhancing nutrient digestibility through stimulation of digestive enzyme secretion, improvement of hepatic and antioxidant enzyme activities, and promotion of enzymes associated with increased heat tolerance (Imbabi et al. 2021; Ragab et al. 2023).

Urea and creatinine are reliable markers for kidney function, with elevated levels in the bloodstream indicating potential renal dysfunction (Embaby et al. 2023). In the present study, NOEO supplementation reduced serum urea and creatinine levels, suggesting its safety and beneficial effects on renal function. This finding is consistent with previous reports indicating that prolonged exposure to high concentrations of EOs does not induce kidney damage or nephritis (Ismail et al. 2023).

Consistent with the biochemical findings, microscopic examination of liver and kidney sections from rabbits in the various treatment groups revealed noticeable differences compared with the control group. Liver and kidney tissues from heat-stressed rabbits exhibited several pathological changes. In contrast, dietary supplementation with NOEO, particularly at 200 and 400 mg/kg, resulted in the most favorable histological outcomes in both liver and kidney tissues compared with lower doses and the control group. These improvements may be associated with enhanced metabolic activity in the NOEO-supplemented groups, reflecting the hepatoprotective and renoprotective effects of the supplement.

Both biochemical assessments and histological evaluations indicated that NOEO supplementation confers hepato-renal protection in heat-stressed rabbits. Similar protective effects have been reported in previous studies (Gupta 2024; Oyinloye et al. 2024). Overall, microscopic examination confirmed that dietary inclusion of NOEO at 100, 200, and 400 mg/kg provided significant histological protection to liver and kidney tissues under HS conditions.

## Conclusion

The present study concluded that heat stress negatively affected the growth performance and overall health of rabbits, leading to elevated oxidative stress. To mitigate these effects, the diet was supplemented with NOEO at 100, 200, and 400 mg/kg to enhance productive performance and improve physiological, immunological, and antioxidant responses. Serum levels of IgM and IgA were significantly higher in rabbits fed NOEO at 200 or 400 mg/kg compared with other groups. Additionally, NOEO supplementation improved serum antioxidant enzyme activities and anti-inflammatory capacities, particularly at the 400 mg/kg inclusion level. These findings highlight the potential of NOEO and its bioactive phytochemical components in modulating intracellular interactions and cellular functions, warranting further investigation at the molecular and cellular levels.

## Data Availability

The data that support the findings of this study are available from the corresponding author, upon reasonable request.
